# Supplementary material for: Inference of genomic landscapes using ordered Hidden Markov Models with emission densities (oHMMed)
Source: BMC Bioinformatics. 2024 Apr 16;25:151. doi: 10.1186/s12859-024-05751-4 (PMC11021005; doi:10.1186/s12859-024-05751-4)
Supplement: Supplementary file 5 — Additional file 5. Extensive documentation of the results of application of oHMMed to the epigenetic marks ATAC, H3K27ac, and H3K27me3 in both 100 kb and 1 kb windows along the human chromosome 1. [file 12859_2024_5751_MOESM5_ESM.pdf]

Supplementary Material, File 5: Inference of Genomic Landscapes using Ordered Hidden Markov Models with Emission Densities (oHMMed)

Claus Vogl[a,b], Mariia Karapetiants[a], Burçin Yıldırım[a,b], Hrönn Kjartansdóttir[a], Carolin Kosiol[c], Juraj Bergman[d], Michal Majka[e], Lynette Caitlin Mikula[c,]

[a] *Department of Biomedical Sciences, Vetmeduni Vienna, Veterinärplatz 1, Vienna, Austria*
[b] *Vienna Graduate School of Population Genetics, A-1210 Vienna, Austria*
[c] *Centre for Biological Diversity, School of Biology, University of St Andrews, St Andrews, Scotland (United Kingdom)*
[d] *Centre for Biodiversity Dynamics in a Changing World (BIOCHANGE) & Section for Ecoinformatics and Biodiversity, Department of Biology, Aarhus University, Aarhus, Denmark*
[e] *Erste Group Bank AG, Vienna, Austria*

1. Segmenting Human Chr1 By Epigenetic Markers

1.1. Selecting the Number of Hidden States in oHMMed

EpiGeneticMark	Window Size (kb)	Number of Chosen States	Criteria
ATAC	100	n=4	Log-Likelihood plateau roughly starts for n=4<br>the means of these states are distinct<br>and convergence behaviour is poor for n>4
	1	n=5	Log-Likelihood plateau clearly starts for n=5<br>the means of these states are distinct<br>and convergence behaviour gets worse for n>5
H3K27ac	100	n=4	Log-Likelihood plateau clearly starts for n=4<br>the means of these states are distinct<br>and convergence behaviour is poor for n>4
	1	n=7	Log-Likelihood plateau roughly starts for n=7<br>the means of these states are distinct<br>and the means for n=8 are no longer distinct
H3K27me3	100	n=4	Log-Likelihood plateau clearly starts for n=4<br>the means of these states are distinct<br>and convergence behaviour poor for n4
	1	n=7	Log-Likelihood plateau roughly starts for n=7<br>the means of these states are distinct<br>and increasing the number of states only further separates the 7th state

Table 1: This table summarises the rationale behind how the most appropriate number of hidden states was chosen for each combination of epigenetic marker and window size; the selected number of hidden states is given in the third column and the reasoning in the fourth. For each data set consisting of counts of epigenetic marks per window, we ran oHHMed with between $n = 2$ and to up to $n = 9$ hidden states and considered the diagnostics for the output of each run according to our own usage recommendations on GitHub[1] in making the decisions.

1.2. Compilation of oHHMed Results for Each Epigenetic Marker

Each table below summarises the most important results for each analysed epigenetic marker. For each of these, we provide the oHMMed-estimated parameters of the emitted poisson-gamma distributions in both 100kb as well as 1*kb* along chromosome 1 for the appropriately chosen number of hidden states. We further show what proportion of windows was assigned to each state, as well as the average number of consecutive windows per the same state that is expected in the data. Subsequent pages show plots of the data with overlayed oHMMed results (the inferred hidden states and the posterior inferred mean).

**ATAC (Poisson-Gamma Emissions)**	**100kb**	**1kb**
Number of Distinct States	4	5
Estimated Alpha	5.527	3.052
Estimated Betas	0.03148 0.02159 0.00386 0.00138	1.79132 1.75006 0.85715 0.09855 0.00655
Estimated Means	175.7 256.3 1434.4 4011.3	1.7040 1.7442 3.5612 30.980 465.91
Estimated Variances	5576.3 1186.0 371400.1 2897027.5	0.95126 0.99663 4.1547 31.436 7110.2
Estimated Transition Rates	0.863 0.137 0.000 0.000<br>0.140 0.441 0.418 0.000<br>0.000 0.411 0.460 0.129<br>0.000 0.000 0.530 0.470	0.008 0.992 0.000 0.000 0.000<br>0.864 0.069 0.067 0.000 0.000<br>0.000 0.076 0.852 0.072 0.000<br>0.000 0.000 0.474 0.332 0.194<br>0.000 0.000 0.000 0.619 0.381
Prop. of Windows per State	0.336 0.281 0.314 0.068	0.193 0.407 0.342 0.043 0.014
Consecutive Windows per State	9.737 1.738 1.911 1.883	1.000 1.960 8.933 1.490 1.625

Table 2: Results for ATAC

**H3K27ac (Poisson-Gamma Emissions)**	**100kb**	**1kb**
Number of Distinct States	4	7
Estimated Alpha	6.635	4.014
Estimated Betas	0.02373 0.01386 0.00448 0.00133	6.64091 1.17448 0.90545 0.47232 0.17884 0.04400 0.00977
Estimated Means	279.9 479.3 1484.3 4991.2	0.60622 3.4180 4.4335 8.4996 22.45 91.27 411.1
Estimated Variances	11794.5 34572.3 331475.4 3748289.1	0.09129 2.9102 4.8965 17.996 125.54 207.44 42091.7
Estimated Transition Rates	0.914 0.086 0.000 0.000<br>0.056 0.684 0.259 0.000<br>0.000 0.306 0.510 0.184<br>0.000 0.000 0.528 0.472	0.721 0.279 0.000 0.000 0.000 0.000 0.000<br>0.051 0.938 0.011 0.000 0.000 0.000 0.000<br>0.000 0.009 0.923 0.068 0.000 0.000 0.000<br>0.000 0.000 0.101 0.816 0.083 0.000 0.000<br>0.000 0.000 0.000 0.218 0.639 0.143 0.000<br>0.000 0.000 0.000 0.000 0.347 0.489 0.164<br>0.000 0.000 0.000 0.000 0.000 0.470 0.530
Prop. of Windows per State	0.228 0.372 0.299 0.101	0.039 0.288 0.345 0.206 0.078 0.033 0.012
Consecutive Windows per State	14.500 3.483 2.102 1.953	4.867 27.839 19.114 6.871 3.070 2.022 2.164

Table 3: Results for H3K27ac

**H3K27me3 (Poisson-Gamma Emissions)**	**100kb**	**1kb**
Number of States	4	7
Estimated Alpha	3.515	7.844
Estimated Betas	0.14202 0.01712 0.00306 0.00110	21.750 4.2349 1.3349 0.55438 0.27348 0.15496 0.08629
Estimated Means	33.838 236.62 1289.5 3194.1	0.36077 1.8528 5.8773 14.152 28.686 50.625 90.909
Estimated Variances	238.3 13821.2 421480.6 2896452.7	0.016587 0.43751 4.4027 25.527 104.89 326.70 1053.5
Estimated Transition Rates	0.425 0.575 0.000 0.000<br>0.053 0.552 0.395 0.000<br>0.000 0.166 0.622 0.212<br>0.000 0.000 0.143 0.857	0.947 0.053 0.000 0.000 0.000 0.000 0.000<br>0.069 0.822 0.108 0.000 0.000 0.000 0.000<br>0.000 0.098 0.772 0.131 0.000 0.000 0.000<br>0.000 0.000 0.113 0.740 0.146 0.000 0.000<br>0.000 0.000 0.000 0.102 0.768 0.130 0.000<br>0.000 0.000 0.000 0.000 0.134 0.812 0.054<br>0.000 0.000 0.000 0.000 0.000 0.241 0.759
Prop. of Windows per State	0.016 0.174 0.331 0.479	0.151 0.113 0.127 0.145 0.213 0.212 0.039
Consecutive Windows per State	1.929 2.474 2.772 7.900	23.701 6.826 5.284 4.645 5.427 7.022 4.811

Table 4: Results for H3K27me3

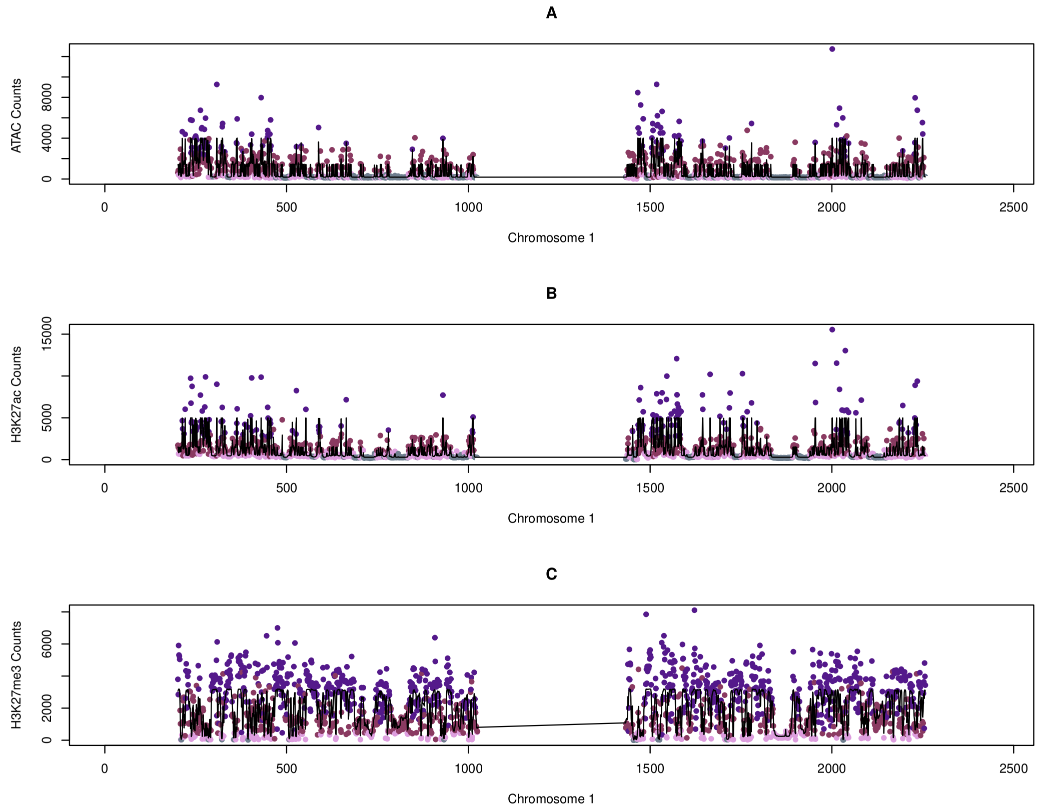

Figure 1: In each of the above panels A-C, the human chromosome 1 is plotted for different oHMMed analyses: In A, the number of ATAC counts per 100kb window are coloured by the oHMMed-inferred state the window has been assigned to. The state with the lowest average count is in grey, and then the states with increasing mean counts are in darkening shades of pink/purple. In panel B, the same plot is shown for H3K27ac, and in panel C, again the same plot for H3K27me3. The gaps on the sides and centre in all panels correspond to the telomere- and centromere-adjacent windows that were removed before the analyses. Note that in all panels, the black lines trace the posterior (inferred) means returned by oHMMed with gamma-poisson emissions. These position-specific posterior means are the sum of estimated means times the respective probabilities of each state, thus combining both estimated mean values and the algorithm's certainty of the assigned state.

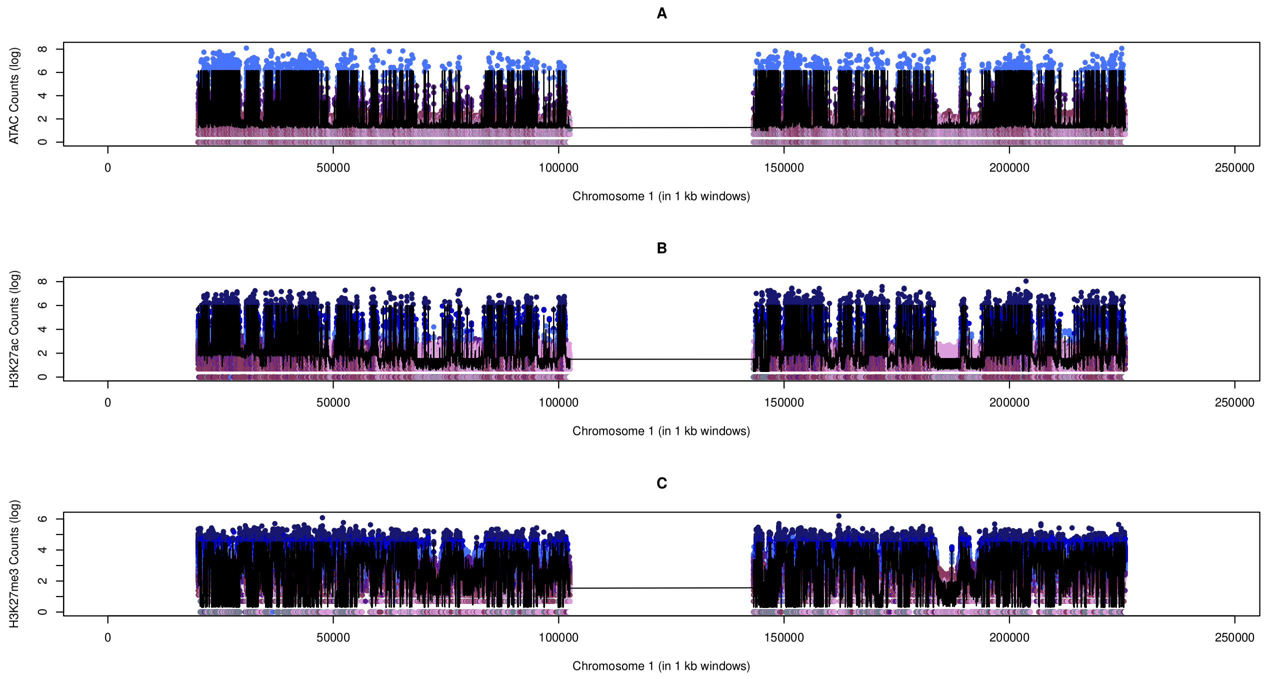

Figure 2: In each of the above panels A-C, the human chromosome 1 is plotted for different oHMMed analyses: In A, the number of ATAC counts per 1kb window are coloured by the oHMMed-inferred state the window has been assigned to. The state with the lowest average count is in grey, and then the states with increasing mean counts are first in darkening shades of pink/purple and then in darkening shades of blue. Importantly, note that the counts have been log-transformed in this case to increase visibility. In panel B, the same plot is shown for H3K27ac, and in panel C, again the same plot for H3K27me3. The gaps on the sides and centre in all panels correspond to the telomere- and centromere-adjacent windows that were removed before the analyses. Note that in all panels, the black lines trace the posterior (inferred) means returned by oHMMed with gamma-poisson emissions. These position-specific posterior means are the sum of estimated means times the respective probabilities of each state, thus combining both estimated mean values and the algorithm's certainty of the assigned state.

References

[1] Mikula, L. (2023). *GitHub - R package ohmmed: Usage Recommendations*.
